# Outcomes of unplanned sarcoma excision: impact of residual disease

**DOI:** 10.1002/cam4.615

**Published:** 2016-03-01

**Authors:** Chris Charoenlap, Jungo Imanishi, Takaaki Tanaka, John Slavin, Samuel Y. Ngan, Sarat Chander, Michelle Maree Dowsey, Chatar Goyal, Peter F.M. Choong

**Affiliations:** ^1^ Department of OrthopaedicsSt. Vincent's Hospital MelbourneVictoriaAustralia; ^2^ Department of Orthopaedics, Faculty of MedicineChulalongkorn University1873 Rama 4 Road, Pathumwan, Bangkok 10400, ThilandThailand; ^3^ Department of PathologySt. Vincent's Hospital Melbourne41 Victoria Parade, Fitzroy 3065VictoriaAustralia; ^4^ Division of Radiation OncologyPeter MacCallum Cancer Centre2 St Andrews Place, East Melbourne 3002VictoriaAustralia; ^5^ Bone and Soft Tissue Sarcoma UnitPeter MacCallum Cancer Centre2 St Andrews Place, East Melbourne 3002VictoriaAustralia; ^6^ Department of SurgeryUniversity of Melbourne29 Regent Street, Fitzroy 3065VictoriaAustralia

**Keywords:** Neoplasm staging, prognosis, residual neoplasm, sarcoma mortality, sarcoma surgery, soft tissue sarcoma, treatment outcome

## Abstract

This study aimed to compare the oncological results between unplanned excision (UE) and planned excision (PE) of malignant soft tissue tumor and to examine the impact of residual tumor (ReT) after UE. Nonmetastatic soft tissue sarcomas surgically treated in 1996–2012 were included in this study. Disease‐specific survival (DSS), metastasis‐free survival (MFS), and local‐recurrence‐free survival (LRFS) were stratified according to the tumor location and American Joint Committee on Cancer Classification 7th edition stage. Independent prognostic parameters were identified by Cox proportional hazard models. Two‐hundred and ninety PEs and 161 UEs were identified. Significant difference in oncological outcome was observed only for LRFS probability of retroperitoneal sarcomas (5‐year LRFS: 33.0% [UE] vs. 71.0% [PE], *P* = 0.018). Among the 142 UEs of extremity and trunk, ReT in re‐excision specimen were found in 75 cases (53%). UEs with ReT had significantly lower survival probabilities and a higher amputation rate than UEs without ReT (5‐year DSS: 68.8% vs. 92%, *P* < 0.001; MFS: 56.1% vs. 90.9%, *P* < 0.001; LRFS: 75.8% vs. 98.4%, *P* = <0.001; amputation rate 18.5% vs. 1.8%, *P* = 0.003). The presence of ReT was an independent poor prognostic predictor for DSS, MFS, and LRFS with hazard ratios of 2.02 (95% confidence interval (CI), 1.25–3.26), 1.62 (95% CI, 1.05–2.51) and 1.94 (95% CI, 1.05–3.59), respectively. Soft tissue sarcomas should be treated in specialized centers and UE should be avoided because of its detrimental effect especially when ReT remains after UE.

## Introduction

Sarcomas are rare malignancies that arise from mesenchymal cells, which occur with an incidence of 2–4 per 100,000 population. It is now accepted that best practice for the management of these tumors belongs in a multidisciplinary setting where the convergence of experience and expertise maximizes the potential for cure or palliation. Despite the widespread availability of treatment guidelines, misdiagnosis and improper surgery still occur among referred sarcomas. Alarmingly, between 37% and 71% of soft tissue sarcomas are subjected to inadvertent surgery prior to referral to specialized tumor centers 1, 2, 3, 4, 5.

Unplanned sarcoma excision has been variously described in the literatures 6, 7, 8, 9, 10. This type of surgery refers to the removal of a mass without prior knowledge of the malignant nature of the tumor and also without the application of planned oncologic margins appropriate for a sarcoma. When referred to a tumor center, re‐excision after unplanned surgery is routinely performed to mitigate the possibility of residual tumor in the previous surgical area 6, 7, 9, 10, 11, 12. In contrast, a planned excision of sarcoma follows appropriate local and systemic staging that includes at least anatomic imaging of the affected part with magnetic resonance imaging (MRI), a chest scan, and tissue biopsy. These investigations are then interrogated by a multidisciplinary team of sarcoma specialists where a consensus opinion is made about the appropriateness of treatment 11.

The outcomes of previously unplanned surgery and the discrepancy between this and the outcomes of planned surgery remain controversial. Authors have reported poor, no difference, or even preferable outcomes in unplanned excision group 3, 4, 5, 7, 10, 12. A key confounder to comparison has been the variations in tumor demographics among these papers. Moreover, unplanned excision patients were frequently treated as a whole group rather than stratified by cancer stage because of the limited numbers. Importantly, stratifying data by residual disease, which is an important outcome predictor, was not always done despite its occurrence in 18–72% of the re‐resection specimen 12, 13, 14, 15.

The aims of this study were to compare the oncological outcome between planned and unplanned excision of soft tissue sarcoma patients after definitive surgery within the same cancer stage, and to clarify the influence of residual disease on oncologic outcomes. The study population consisted of patients with localized sarcoma that was appropriate for treatment with curative intent.

## Materials and Methods

### Study design

St. Vincent's Hospital Melbourne and Peter MacCallum Cancer Centre East Melbourne, where this study was conducted, are metropolitan specialist hospitals which form the conjoint sarcoma service, a national teritary referral center for sarcoma care. The research protocol was approved by the institutional human research ethics committee (HREC number: QA 012/15). Patients meeting the inclusion criteria below were identified from a prospectively compiled institutional connective tissue tumor database. All patient medical records, imaging and pathological reports were reviewed and updated with patient status information from patient records at hospitals and the Cancer Council of Victoria, which is a not‐for‐profit organization aiming to reduce the impact of cancer and has access to death information of all the registered sarcoma patients.

### Inclusion criteria and definition of unplanned excision

The inclusion criteria were soft tissue sarcomas surgically treated in 1996–2012, no lymph node or distant metastasis prior to definitive resection, and at least a 2‐year follow‐up period if the absence of oncological event. Unplanned excision (UE) was defined as the removal of sarcoma without concern of an appropriate surgical margin due to a mistaken diagnosis of benign disease or the absence of histopathological confirmation, including inappropriate partial resection or open biopsy outside using wrong biopsy tract or having widespread contamination that would be otherwise preventable. Patients, who had no prior surgery or were referred after appropriate biopsy, or who underwent primary resection in our institution were allocated to the planned excision (PE) group. Residual tumor (ReT) was declared if sarcoma tissue was microscopically found in the re‐excision specimen of a UE case.

### Definition of other variables in this study

Age was determined as the age of patient at the diagnosis date. Tumor size was determined by measuring the largest diameter of tumor mass on any axis from magnetic resonance images at the first presentation before preoperative radiotherapy. If the imaging was unavailable for the UE group, the pathological reports of the first operation were used instead. Tumor grade was determined according to the Fédération Nationale des Centers de Lutte le Cancer (FNCLCC) grading system 16. Patients were staged using the AJCC (American Joint Committee on Cancer) classification 7th edition 17. Tumors superficial to the deep fascia were classified as superficial, and those deep to or engaged in the deep fascia were classified as deep. Surgical margin were classified according to the Enneking system 18. A microscopically positive margin was declared if surgical margin was identified at the inked margin of definitive resection specimen. In this study, all cases with microscopically positive margin were categorized as “intralesional margin,” regardless of intra‐operative or macroscopic findings.

The disease‐specific survival (DSS) time was calculated from the date of diagnosis to the date of death from disease. Mortality from other causes was censored. Metastasis‐free survival (MFS) time and local‐recurrence‐free survival (LRFS) time were counted from the date of definitive surgery to the date that distant metastasis at any site and recurrences at or near primary tumor location were recorded, respectively. Local recurrence and distant metastasis were confirmed by biopsy or resection unless the recurrence or metastasis was multiple or clinically obvious.

The amputation rate was calculated by dividing the number of eventually amputated cases, including cases that initially underwent limb‐salvage surgery but ultimately required amputation, by the number of cases in each category.

### Treatment and follow‐up protocol at our sarcoma service

Radiotherapy consisted of 50.4 Gy delivered by external beam in divided doses over 28 sessions was applied to the majority of soft tissue sarcoma cases, whether UE or PE, except for certain small low‐grade cases or those in which amputation was planned at the first presentation. The indication of chemotherapy was determined at the institutional multidisciplinary meeting. Adjuvant chemotherapy was considered for specific histotypes, such as Ewing's sarcoma, rhabdomyosarcoma, extraskeletal osteosarcoma, and synovial sarcoma.

After definitive surgery, the specimen was examined macroscopically and microscopically and reviewed at our multidisciplinary meeting. The seven patients with intralesional surgical margin in this study were closely followed up clinically and radiologically without immediate additional resurgery or radiotherapy.

Patients were followed at least for 8 years after surgery including clinical and radiographic examination every 3–4 months in the first 2 years, 6 months for the next 2 years, and then every year.

### Statistical methods

Differences in categorical data were analyzed using chi‐square test or Fisher's exact test. Student's *t*‐test was used to compare continuous variables. The oncological outcomes between PE and UE groups, including 5‐year DSS, MFS, and LRFS probability, were compared among each site group (extremity & trunk, head & neck, and retroperitoneal) using Kaplan–Meier method and log‐rank test. Intermediate malignancy tumors were evaluated only for LRFS due to their low potential of metastasis and mortality. Soft tissue sarcomas of extremity and trunk were selected for analyzing the effect of ReT on oncological outcomes. Possible prognostic variables chosen for univariate log‐rank test and Cox proportional hazard multivariate analysis with stepwise elimination method were gender (female or male), age (≤60 years or >60 years), tumor location (extremity or trunk), tumor size (≤5 cm or >5 cm), tumor depth (superficial or deep), FNCLCC grade (grades 1, 2, or 3), surgical margin (radical/wide or marginal/intralesional), received adjuvant radiotherapy (with or without adjuvant radiotherapy), received adjuvant chemotherapy (with or without adjuvant chemotherapy), and residual tumor status (PE, UE with ReT, or UE without ReT). A *P*‐value of less than 0.05 was regarded as statistically significant. All statistical analyses were computed using SPSS version 17 (SPSS Inc., Chicago, USA).

## Results

### Patients included in this study

Five hundred and thirty‐one surgically treated soft tissue sarcomas were identified from the database. Forty patients had antecedent metastasis, 25 patients were properly treated with wide surgical margin and referred after recurrence, nine cases had follow‐up period shorter than 2 years, and six patients had no tumor size. These 80 cases were excluded from this study.

**Figure 1:**
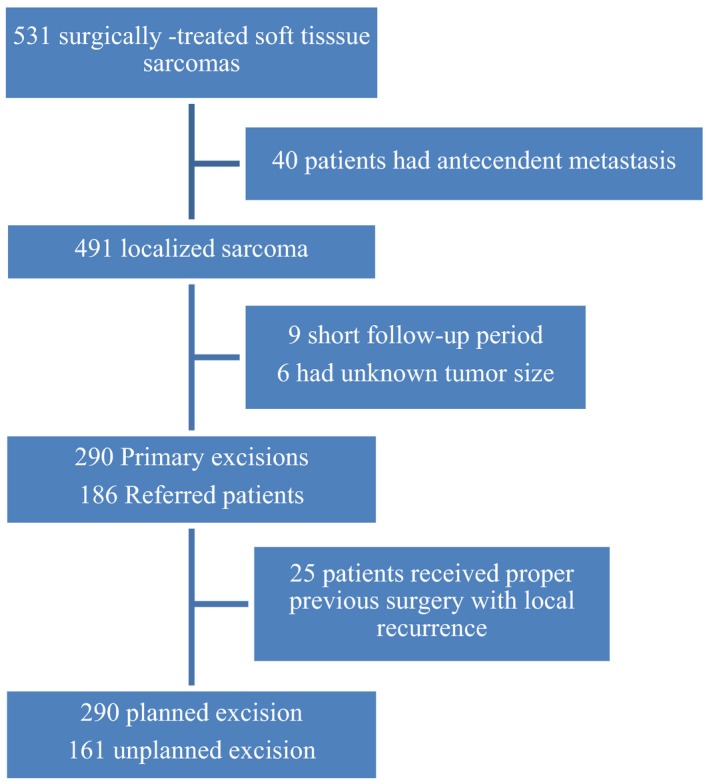
Flow diagram of patients included in this study.

A total of 451 patients were enrolled comprising 290 PEs and 161 UEs, including six inappropriate partial excision or open biopsy in UE (Fig. 1, Table 1). There were 251 males with a male: female ratio of 1.3:1. The average age was 54.6 years (range, 15–95). The median follow‐up time for survivors was 72.6 months.

**Table 1:** Patient characteristics of PE and UE, *n* = 451

Characteristic	PE	UE	*P* a
	No. (%)	No. (%)	
Total	290	161	
Gender			
Male	157 (54.1)	94 (58.4)	0.384
Age, year			
≤60	161 (55.5)	90 (56.9)	0.937
Site			
Extremity	241 (83.1)	131 (81.4)	0.247
Trunk	29 (10)	22 (13.7)	
Head and Neck	6 (2.1)	3 (1.9)	
Retroperitoneal	14 (4.8)	5 (3.1)	
Histologic subtype			
Malignant			0.157
UPS	107 (36.9)	61 (37.9)	
Liposarcoma	48 (16.5)	13 (8.1)	
Synovial sarcoma	23 (7.9)	18 (11.2)	
Leiomyosarcoma	23 (7.9)	20 (12.4)	
Myxofibrosarcoma	14 (4.8)	6 (3.7)	
Other	40 (13.8)	32 (19.9)	
Intermediate malignancy			0.007
Well‐differentiated liposarcoma	32 (11)	6 (3.7)	
Solitary fibrous tumor	3 (1)	3 (1.9)	
Myofibroblastic sarcoma	0 (0)	2 (1.2)	
Size, cm			
≤5	74 (25.5)	92 (57.1)	<0.001
Depth			
Superficial	41 (14.1)	75 (46.6)	<0.001
FNCLCC grade^b^			
1	23 (9)	40 (26.7)	<0.001
2	80 (31.4)	40 (26.7)	
3	152 (59.6)	70 (46.7)	
AJCC stage^b^			
I	23 (9)	40 (26.7)	<0.001
II	109 (42.7)	76 (50.7)	
III	123 (48.2)	34 (22.7)	
Surgical margin			
Intralesional/marginal	4/19 (8)	2/6 (5)	0.612
Adjuvant radiotherapy			
Yes	258 (89)	136 (84.5)	0.169
Adjuvant chemotherapy			
Yes	12 (4.1)	10 (6.2)	0.327

PE, Planned excision; UE, Unplanned excision; UPS, Undifferentiated pleomorphic sarcoma; FNCLCC, Fédération Nationale des Centres de Lutte le Cancer; AJCC, American Joint Committee on Cancer.

a Chi‐square test for comparison between PE and UE.

b Malignant tumor cases only, *n* = 405.

Tumors were located in 90 upper limbs, 282 lower limbs, 51 superficial trunk walls, 17 retroperitoneal areas, and 11 head & neck regions. There were 405 malignant soft tissue tumors including 168 undifferentiated pleomorphic sarcomas (UPSs), 61 liposarcomas (45 myxoid/round cell, 12 pleomorphic, and four dedifferentiated liposarcomas), 41 synovial sarcomas, 43 leiomyosarcomas, 20 myxofibrosarcomas, 13 malignant peripheral nerve sheath tumors, and 59 other malignant soft tissue tumors. Forty‐six intermediate malignancy tumors were 38 well‐differentiated liposarcomas, six solitary fibrous tumors, and two myofibroblastic sarcomas. The mean tumor size was 8.6 cm (range, 1–40). The margins employed in the initial surgery of UEs included 110 intralesional, 51 marginal margins.

Three hundred and ninety‐four of 451 patients (87.4%) received adjuvant radiotherapy (131 preoperative and three postoperative radiotherapy for UE; 250 preoperative and eight postoperative radiotherapy for PE). Thirty‐eight patients received chemotherapy (22 neo‐adjuvant/adjuvant, 16 palliative after local or systemic recurrence).

### Comparison between UE and PE

UE group more often involved tumors that were smaller than those from planned resections (mean 6.2 cm vs. 9.9 cm; *P* < 0.001). The majority of unplanned tumor resections involved tumors that were 5 cm or less (57.1% vs. 25.5%; *P* < 0.001). Tumors subjected to UE were more often superficial as compared to planned resections (46.6% vs. 14.1%; *P* < 0.001). Tumors subjected to unplanned excisions were more often lower grade and stage than tumors excised as planned resections (Table 1).

Wide or radical margin was achieved for re‐excision in 95% of UE patients and it was similar to 92.4% of PE cases (*P* = 0.612). Despite UE being associated with smaller, more superficial and lower‐grade tumors, the proportion of extremity amputations for UE and PE were comparable (10.7% vs. 9.4%, *P* = 0.719). Requirement of soft tissue flap reconstructions to rebuild the operative defect was comparable between PE and UE (20.4% vs. 23.9%, *P* = 0.384).

**Figure 2:**
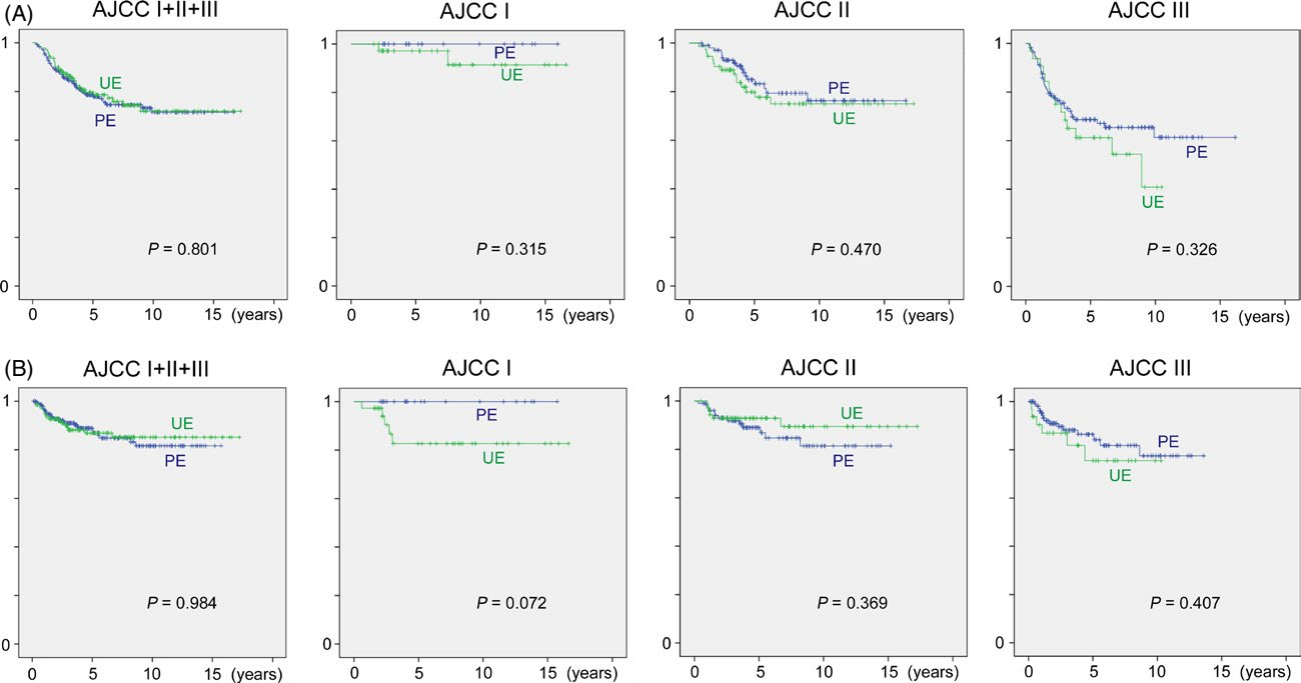
(A) Disease‐specific survival and (B) local‐recurrence‐free survival of planned excision (PE) and unplanned excision (UE) of extremity and trunk sarcoma.

For extremity and trunk tumors, DSS for specific AJCC stage tended to be lower for UE, and a similar trend was observed in stages I for MFS and stage I, III for LRFS (Table 2 and Fig. 2). DSS in head and neck sarcoma was lower in UE group; however, the probability of MFS and LRFS probability was equal. Retroperitoneal tumors had the worst DSS rate than the other sites and LRFS of UE in this area was significantly lower than PE (*P* = 0.018). Percentage of LRFS between PE and UE was similar in intermediate malignancy tumors.

**Table 2:** Comparison of oncologic outcome between planned excision and unplanned excision

Outcome	PE (CIs)	UE (CIs)	*P* a
5‐year DSS (%)			
Extremity and trunk	78.6 (73.1–84.1)	79.7 (72.6–86.8)	0.801
Stage I	100	97.1 (91.6–100)	0.315
Stage II	84.9 (77.3–92.5)	79.9 (69.9–89.9)	0.470
Stage III	68.7 (59.7–77.7)	61.2 (44–78.4)	0.326
Head and neck	100	75 (32.5–100)	0.386
Retroperitoneal	59.3 (23–95.6)	33.3 (0–86.6)	0.187
5‐year MFS (%)			
Extremity and trunk	65.9 (59.6–72.2)	72.7 (64.9–80.5)	0.088
Stage I	95 (85.4–100)	80.3 (65.6–95)	0.183
Stage II	73.7 (64.7–82.7)	74.9 (64.3–85.5)	0.909
Stage III	53.2 (43.8–62.6)	58 (39.6–76.4)	0.573
Head and neck	100	100	−
Retroperitoneal	66.7 (35.9–97.5)	100	0.351
5‐year LRFS (%)			
Extremity and trunk	88.9 (84.4–93.4)	86.9 (80.8–93)	0.984
Stage I	100	82.7 (68.8–96.6)	0.072
Stage II	89 (82.5–95.5)	92.9 (87–98.8)	0.369
Stage III	86.4 (79–93.8)	75.5 (57.3–93.7)	0.407
Head and neck	80 (44.9–100)	80 (44.9–100)	0.937
Retroperitoneal	71.1 (35.8–100)	33 (0–86.6)	0.018
Intermediate tumor	97.5 (91.6–100)	100	0.509

PE, Planned excision; UE, Unplanned excision; CIs, 95% confidence intervals; DSS, Disease‐specific survival; MFS, Metastasis‐free survival; LRFS, Local‐recurrence‐free survival; −, No event occurred in both groups.

a Log‐rank test.

### ReT in re‐excision specimen

ReT was presented in 88, of 161 UEs re‐excision specimens (54.7%), including 15 macroscopically ReT‐negative but microscopically ReT‐positive cases. Seventy‐five of 142 (52.8%) UEs of extremity and trunk had residual disease (Table 3). ReT was more often found in older age and larger tumor size than the nonresidual group.

**Table 3:** Background of extremity and trunk UE cases with and without residual tumor, *n* = 142

Characteristic	ReT (+)	ReT (−)	*P*
	No. (%)	No. (%)	
Total	75	67	
Gender			
Male	39 (52)	43 (64.2)	0.174^b^
Age, year			
≤60	36 (48)	46 (68.7)	0.017^b^
Site			
Extremity	65 (86.7)	46 (68.7)	0.813^b^
Trunk	10 (13.3)	21 (31.3)	
Size, cm			
≤5	37 (49.3)	47 (70.1)	0.016^b^
Depth			
Superficial	31 (41.3)	38 (56.7)	0.092^b^
FNCLCC grade			
1	17 (22.7)	20 (29.9)	0.050^a^
2	16 (21.3)	23 (34.3)	
3	42 (56)	24 (35.8)	
AJCC stage			
I	17 (22.7)	20 (29.9)	0.005^a^
II	33 (44)	40 (59.7)	
III	25 (33.3)	7 (10.4)	
Surgical margin			
Intralesional/marginal	6 (8)^c^	1 (1.5)	0.120^b^
Adjuvant radiotherapy			
Yes	60 (80)	61 (91)	0.096^b^
Adjuvant chemotherapy			
Yes	6 (8)	3 (4.5)	0.500^b^

UE, Unplanned excision; ReT, Residual tumor; FNCLCC, Fédération Nationale des Centres de Lutte le Cancer; AJCC, American Joint Committee on Cancer.

a Chi‐square test.

b Fisher's exact test.

c Including four microscopically positive margin cases.

Amputation rate of extremity sarcomas was significantly different when residual disease was considered; 12 of 65 UEs with ReT lost their limbs as compared to 1 of 57 UEs without ReT (18.5% vs. 1.8%, *P* = 0.003).

**Figure 3:**
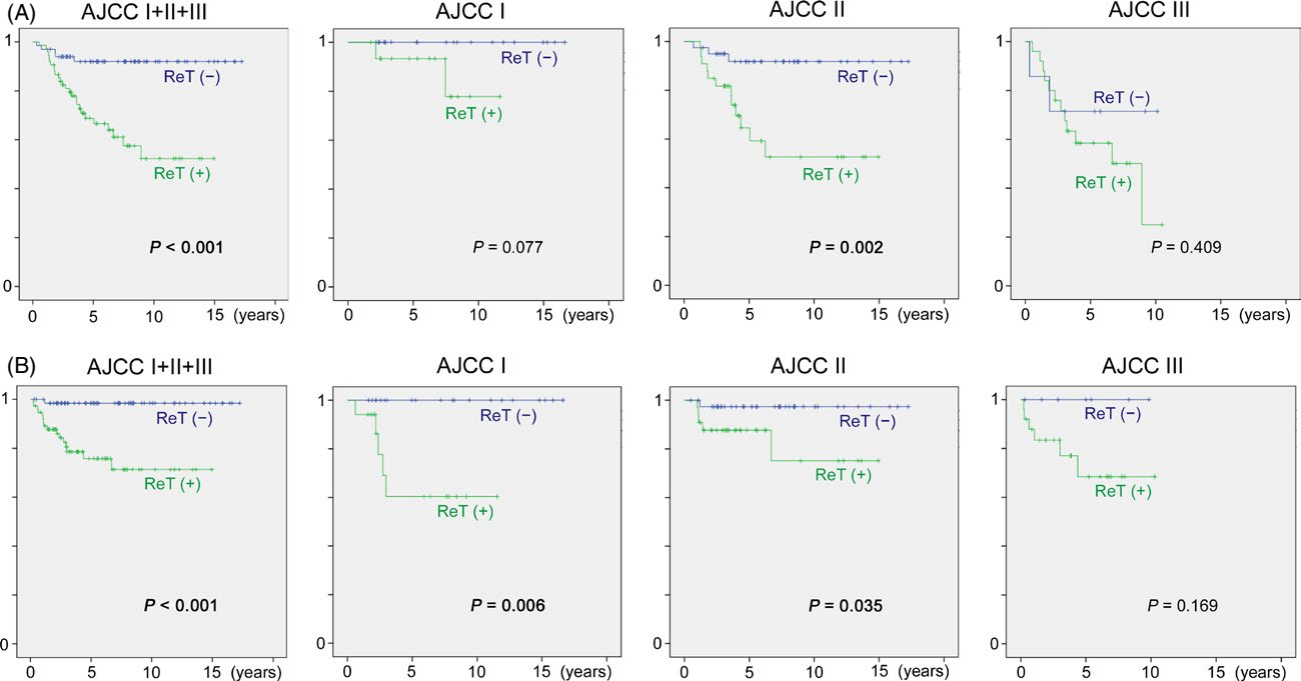
(A) Disease‐specific survival and (B) local‐recurrence‐free survival of unplanned excision with residual tumor (ReT (+)) and without residual tumor (ReT (−)) in extremity and trunk sarcoma.

UE with ReT had significantly lower survival than UE without ReT in stage I (LRFS: *P* = 0.006; MFS: *P* = 0.045) and stage II (LRFS: *P* = 0.035, MFS: *P* = 0.008 and DSS: *P* = 0.002) (Table 4, Fig. 3).

**Table 4:** Comparison of oncologic outcomes between PE and UE with and without ReT in extremity and trunk sarcomas

Outcome	PE (CIs)	UE (CIs)	UE (CIs)	*P* a
		ReT (+)	ReT (−)	
5‐year DSS (%)				
All	78.6 (73.1–84.1)	68.8 (57.4–80.2)	92 (85.1–98.9)	<0.001
Stage I	100	93.3 (80.8–100)	100	0.077
Stage II	84.9 (77.3–92.5)	64.6 (46.2–83)	91.8 (82.8–100)	0.002
Stage III	68.7 (59.7–77.7)	58.5 (38.5–78.5)	71.4 (37.9–100)	0.409
5‐year MFS (%)				
All	65.9 (59.6–72.2)	56.1 (43.6–68.6)	90.9 (84–97.8)	<0.001
Stage I	95 (85.4–100)	95 (85.4–100)	62.9 (36–89.8)	0.045
Stage II	73.7 (64.7–82.7)	55.7 (36.5–74.9)	89.9 (80.5–99.3)	0.008
Stage III	53.2 (43.8–62.6)	51.5 (30.5–72.5)	83.3 (53.5–100)	0.163
5‐year LRFS (%)				
All	88.9 (84.4–93.4)	75.8 (64.6–87)	98.4 (95.3–100)	<0.001
Stage I	100	60.4 (33–87.8)	100	0.006
Stage II	89 (82.5–95.5)	87.7 (76.3–99.1)	97.4 (92.3–100)	0.035
Stage III	86.4 (79–93.8)	68.5 (45.8–91.2)	100	0.169

PE, Planned excision; UE, Unplanned excision; CIs, 95% confidence intervals; ReT, Residual tumor; DSS, Disease‐specific survival; MFS, Metastasis‐free survival; LRFS, Local‐recurrence‐free survival.

a Log‐rank test between UE with and without ReT.

### Prognostic factors

Cox proportional hazards analysis revealed the following predictors as independent adverse predictors: size greater than 5 cm, FNCLCC grades 2 and 3, and UE with ReT (for DSS), size greater than 5 cm, FNCLCC grade 3, deep location and UE with ReT (for MFS), and ReT status (for LRFS) (Table 5). The strongest negative predictor for LRFS was ReT status, and for MFS and DSS was tumor grade.

**Table 5:** Prognostic factors for DSS, MFS, and LRFS of extremity and trunk sarcomas

Prognostic factor	Reference	HR (95% CI)	*P*
DSS			
Size > 5 cm	Size ≤ 5 cm	2.39 (1.39–4.11)	0.002
FNCLCC 2	FNCLCC 1	5.58 (1.30–23.91)	0.020
FNCLCC 3	FNCLCC 1	6.47 (1.55–26.94)	0.010
UE with ReT	PE	2.02 (1.25–3.26)	0.004
MFS			
Size > 5 cm	Size ≤ 5 cm	1.86 (1.18–2.93)	0.007
FNCLCC 3	FNCLCC 1	2.92 (1.33–6.40)	0.008
Deep location	Superficial location	1.74 (1.03–2.95)	0.040
UE with ReT	PE	1.62 (1.05–2.51)	0.030
LRFS			
UE without ReT	PE	0.11 (0.02–0.79)	0.028
UE with ReT	PE	1.94 (1.05–3.59)	0.034

DSS, Disease‐specific survival; MFS, Metastasis‐free survival; LRFS, Local‐recurrence‐free survival; HR, Hazard ratio; CI, Confidence interval; FNCLCC, Fédération Nationale des Centres de Lutte le Cancer; UE, Unplanned excision; ReT, Residual tumor; PE, Planned excision.

## Discussion

Soft tissue sarcomas are rare and this may explain the high incidence of inadvertent excision. While most general practitioners and surgeons may only see a limited number of soft tissue sarcomas in a lifetime, they may see a considerable number of small and superficial benign entities such as lipoma and schwannoma. In this regard, this study identified that the proportion of small and superficial tumors was significantly higher in unplanned excisions as compared to planned excisions. The main consequences for patients who undergo inadvertent excision is the requirement of further surgery, the delay in appropriate treatment, the financial cost of further care, and the impact on local and systemic outcomes 12, 19, 20, 21.

A worrisome finding is that despite available guidelines and formal medical training, 33% of referred patients to our institution occurred after inadvertent excision. This is similar to other reports where prereferral surgical intervention may be as high as 70% 1, 2, 3, 4, 5, 9, 12, 14, 22. The local consequences of inadvertent resection are either contamination of surrounding structures or the presence of residual disease. These two may dramatically alter the subsequent local management of disease to avoid local recurrence of disease.

Residual disease was detected in at least 55% of all patients in our series who were referred in for subsequent surgical management after unplanned excisions. This is similar to that reported in the literature 13. At times, the finding of positive margin may herald this possibility of residual disease. In our study, the incidence of positive margins in previous surgery before referral was 87%. The literature has reported a similar incidence with a range that can be as high as 82% 23. What is compelling about our findings and others, of the high incidence of residual tumor and positive margin surgery following inadvertent resection is that these tumors are usually smaller (≤5 cm) and superficial to the deep fascia in comparison to tumors primarily treated at a specialist center where they are larger (>5 cm) and deep to the deep fascia. What unplanned excisions invoke is the potential for impacting treatment and outcomes by significantly upgrading local surgical treatment in a tumor that would normally be easily treated by a specialist center 11, 14, 24. What such upgrading may require is the use of soft tissue flap reconstructions or amputation to control local disease. Almost 1/5 of all patients who were referred after inadvertent excision with residual tumor required amputation for local control in our series. This was over twice the amount as compared to patients with planned excisions. Others have also reported the need for amputation to control local disease for referrals after inadvertent excision and specifically for the management of extensive soft tissue complications 24.

What remains controversial is the impact of UEs on survival outcomes. The literature remains ambivalent with some studies reporting poorer rates of local control after unplanned excisions, while others have reported paradoxically the opposite 7, 10, 12. In our study, apart from retroperitoneal tumor, no significant difference in LRFS between UE and PE was found (Table 2 and Fig. 2). However, in the cohort of extremity and trunk sarcomas, a relatively strong tendency for poorer LRFS was observed at AJCC stage I (*P* = 0.072), which would otherwise enjoy the best outcomes after appropriate management because of their small size and lower grade. This result may demonstrate that small soft tissue sarcomas do occur in significant proportions but appropriate diagnosis of these is crucial for good oncological outcomes. In contrast, the results of stages II and III may mean that wider resection including possible contaminated area or amputation can reduce the risk of local recurrence after UE to the same level as in PE.

By contrast with the poorer local control of UE reported by many papers, significantly poor patient survival for UE, overall survival or DSS, has never been published. In our study, even after stratification according to the AJCC stage, despite favorable DSS of PE over UE, there was no statistical significance at each stage of extremity and trunk sarcomas (*P* = 0.315–0.470). Our result is consistent with the past literature, but further investigation to compare UE with PE with a larger number of cases at each stratified group is required for verification.

A number of authors have highlighted the significance of residual tumor on survival 8, 12, 15. We have shown that ReT‐positive group had significantly worse outcomes for stages I and II. Even for stage III, a low statistical power due to small number of cases (only 25 ReT‐negative and 8 ReT‐positive cases at stage III) can be the main reason for no significance. Also, by Cox analysis, ReT‐positive status was revealed as an independent adverse predictor of local control and disease‐specific survivals, along with larger size and higher grade. This is a critical finding in light of the fact that 55% of all referred patients who have experienced UE had residual tumor. This means that approximately a half of UE patients fall into a category in which significantly poorer outcomes are expected. However, the interpretation of this finding should be careful because ReT status can be a confounding factor that can be affected by various other factors. Although poor quality of preceding UE procedure is thought to be mainly responsible for ReT‐positive status, it may partially represent invasive nature or infiltration of tumor. In other words, some of the ReT positivity may have been due to tumor nature rather than UE procedure itself.

There were some limitations of the current study. Firstly, the research design was retrospective review which may be affected by selection bias. Secondly, the statistical power is reduced when stratifying for subgroup analysis due to the limited number of patients in each group. This may be more pertinent with regard to AJCC stage III (statistical power = 0.4). Lastly, tumor staging depended on either MRI or from the pathology report.

Our study highlights the importance of appropriate sarcoma patient management. The fact that even excision of small and superficial tumors, which are often targeted by nonexperts, may result in significantly inferior survival and outcomes provides compelling evidence that all soft tissue lumps should undergo anatomic imaging and be considered for biopsy prior to excision by expert teams.

## Conflict of Interest

The authors made no disclosures.
